# Sequencing-based variant detection in the polyploid crop oilseed rape

**DOI:** 10.1186/1471-2229-13-111

**Published:** 2013-08-06

**Authors:** Rachel Wells, Martin Trick, Fiona Fraser, Eleni Soumpourou, Leah Clissold, Colin Morgan, Jérôme Pauquet, Ian Bancroft

**Affiliations:** 1 John Innes Centre, Norwich Research Park, Norwich NR4 7UH, UK; 2 Present address: The Genome Analysis Centre, Norwich Research Park, Norwich NR4 7UH, UK; 3 BIOGEMMA S.A.S., Chemin de Panedautes, Domaine de Sandreau, 31700, Mondonville, France; 4 Present address: Department of Biology, University of York, Wentworth Way, Heslington, York YO10 5DD, UK

**Keywords:** SNP, Mutation, Polyploid, Crop

## Abstract

**Background:**

The detection and exploitation of genetic variation underpins crop improvement. However, the polyploid nature of the genomes of many of our most important crops represents a barrier, particularly for the analysis of variation within genes. To overcome this, we aimed to develop methodologies based on amplicon sequencing that involve the incorporation of barcoded amplification tags (BATs) into PCR products.

**Results:**

A protocol was developed to tag PCR products with 5’ 6-base oligonucleotide barcode extensions before pooling for sequencing library production using standard Illumina adapters. A computational method was developed for the de-convolution of products and the robust detection and scoring of sequence variants. Using this methodology, amplicons targeted to gene sequences were screened across a *B. napus* mapping population and the resulting allele scoring strings for 24 markers linkage mapped to the expected regions of the genome. Furthermore, using one-dimensional 8-fold pooling, 4608 lines of a *B. napus* mutation population were screened for induced mutations in a locus-specific amplicon (an orthologue of *GL2.b*) and mixed product of three co-amplified loci (orthologues of *FAD2*), identifying 10 and 41 mutants respectively.

**Conclusions:**

The utilisation of barcode tags to de-convolute pooled PCR products in multiplexed, variation screening via Illumina sequencing provides a cost effective method for SNP genotyping and mutation detection and, potentially, markers for causative changes, even in polyploid species. Combining this approach with existing Illumina multiplexing workflows allows the analysis of thousands of lines cheaply and efficiently in a single sequencing run with minimal library production costs.

## Background

By 2050 the world population is predicted to reach 9.1 billion, 34% higher than today, and to meet global demand will require an increase in food production of 70% [1]. Expansion of cultivation area is an increasingly unlikely and costly solution [2] and though gains can be achieved through improved agronomic management [3], current advances in genetics and genomics offer many opportunities not only for crop genetic research but also technology assisted cultivar development.

Fundamental research in “model” plant species such as *Arabidopsis thaliana*, the first plant for which a high quality genome sequence was obtained [4], has led to the identification of genes implicated in the control of many aspects of plant development, metabolism and environmental interactions. However, many of our most important crops, including bread wheat, potato, cotton and sugarcane are polyploids and many agronomic traits in crop species are typically controlled by multiple genes or quantitative trait loci (QTL). Cost effective, efficient, high density marker platforms are therefore paramount in facilitating comparative genetic mapping and the identification of candidate genes and favourable alleles via QTL analysis, association studies or mutation screening. Ultimately these platforms will also assist in the successful incorporation of this genetic variation into breeding programs and the development of superior cultivars, especially when introgressing traits with low heritability (i.e. where environmental effects are relatively large).

Commonly used molecular markers such as amplified fragment length polymorphisms (AFLP) [5], restriction length polymorphisms (RFLP) [6] and simple sequence repeats (SSR) [7] have generally been assayed on low throughput agarose or acrylamide gel electrophoresis to detect size or conformation polymorphisms. Multiplexing using capillary sequencing technology has been achieved by the incorporation of fluoro-labels to the PCR products. Schuelke (2000) developed a universal M13 tail, three primer marker system allowing the fluoro-labels to be used in combination with many different sets of specific primers and multiplexed in the most efficient permutations, reducing genotyping costs [8], however as an assay, they are still expensive per data point.

More recent developments in array technologies allow the screening of thousands of markers on an individual in a single hybridisation assay. Diversity array technology (DArT) markers provide relatively low cost genotyping without prior sequence information and have been widely used for genotyping in crops [9], [10]. More recently, single nucleotide polymorphisms (SNP) have become the predominant marker type for crop improvement. Illumina’s GoldenGate [11], one of the most popular technologies for analysis of SNP markers, is based on BeadArray/Chips and offers genotyping with low costs per data point when large numbers of markers are assayed in parallel, but unlike DArT requires previous sequence knowledge. Genomic DNA is bound to paramagnetic particles and an oligonucleotide pool is added that contains allele-specific primers with universal priming sites and SNP-linked locus-specific primers carrying bead address sequences. Following extension and ligation, an allele-specific fluoro-labelled PCR is performed and the resulting products hybridised against beads carrying locus address sequences arrayed on a glass slide. Genotyping is then performed by reading the fluorescence at each address. Fairly large collection of samples (maximum 384) can be genotyped for a customized pool of markers, up to 1536 SNPs, simultaneously [9]. Building on this technology, Illuminas Infinium array also uses bead chips but is based on random amplification of fragments across the whole genome [12]. Each chip is divided into between 4 and 24 sections, each containing between 1 million and 90,000 bead sites [13], and can either be used to genotype hundreds of thousands of SNPs via loading different bead pools/section for a single sample, or alternatively several thousands SNPs by loading different samples in each section. These high throughput SNP genotyping technologies are efficient for scoring a large number of markers against relatively few individuals. However, for marker-led breeding, the requirement is more typically for the analysis of a few markers on thousands of individuals. As advances in our ability to identify the precise molecular bases of allelic variation continue to improve, the emphasis will shift from the development of linked anonymous markers to causative variation within genes, presenting particular problems in polyploid species, which will contain multiple very closely related genes that would interfere with assays.

High throughput second generation sequencing approaches, even the benchtop platforms of which can produce up to 1.6 Gb of sequence per run [14], have revolutionised our approach to undertaking genetic analyses, with Genotyping by Sequencing (GBS) becoming increasingly popular. Step changes have been made using the Illumina sequencing technology for SNP discovery, linkage mapping and association genetics, even for polyploid species for which genome sequences are not available [15-17]. Although these sequencing technologies are extremely cost-effective for producing large quantities of sequence data from individual samples, they are not well-suited to producing modest quantities of sequence data from large numbers of samples. This is because the sequencing library preparation is expensive. Attempts to circumvent this problem have to date focussed on various strategies of indexing or barcoding based on variant adapters, enabling the multiplexing of multiple samples per run of the instrument, and has been used for the analysis of modest numbers of amplicons in plants as well as in human genetic studies [18-20]. The length and design constraints of the sequencing adapters, however, make such an approach too expensive for sequence variant identification in thousands of PCR products across large panels of target loci in crop species.

Current approaches to detecting simple sequence variation in mutation screens relies on methods such as Targeting Induced Local Lesions IN Genomes (TILLING) [21], which identifies sequence variants in locus-specific PCR products from pooled plants, representing coding regions of genes and typically employing an endonuclease (Cel1) that cleaves at mismatches in heteroduplex DNA [22,23] or conventional Sanger sequencing of a target amplicon. These techniques, though well established, rely on the development of locus-specific amplicons within genes, which is often problematic in polyploid crops. TILLING has been performed on mixed amplicons with very high level of conservation as demonstrated by Wang *et al.* (2008) who identified mutants in a mixed amplicon of the two *BnFAE1* genes, which show a sequence similarity of 98.6% [24]. However, screening such mixed amplicons by the conventional TILLING approaches is rarely feasible as heteroduplex cleavage products are produced at every mismatch position throughout the amplicon, obscuring the much rarer (and weaker) signals from mutations induced in the population.

In the present study we aimed to develop a fast, robust and low-cost technology for SNP genotyping even within genes, so has the potential for deployment with “perfect” markers representing the causative changes underlying allelic variants. We based the methodology on Illumina NGS platforms using as a representative polyploid the globally important oil crop, oilseed rape; species *Brassica napus*. Oilseed rape is a recently formed polyploid believed to have arisen less than 10,000 years ago following hybridisation events between the diploid *Brassica* species *B. rapa* (A genome) and *B. oleracea* (C genome). Comparative mapping between the diploids and *A. thaliana* suggest that the *Brassica* genomes are composed of three rearranged variants of an ancestral genome [25], further complicating *B. napus* genome architecture. Because of this complexity, locus-specific PCR amplicons cannot always be obtained (especially for homoeologous loci, i.e. corresponding loci in the A and C genomes), limiting the number of sites in the genome that can be screened for sequence variation by conventional approaches. We developed an approach to incorporate oligonucleotide barcodes into PCR products, based on a well-established method for fluorescent labelling [26]. The barcoded amplicons can be pooled from many separate reactions prior to sequencing library production and sequenced using standard Illumina workflows. Computational analysis of variants based on that developed for SNP discovery in polyploids [16] were utilised for SNP assay in mixed amplicons (comprising homoeologous pairs of loci). For the study we chose loci close to members of two important fatty acid biosynthesis gene families: *BnaFAD2* and *BnaSAD3*. *BnaFAD2* has four copies in *B. napus* located on linkage groups A1 (*BnaA.FAD2.b-like*), A5 (*BnaA.FAD2.a*), C1 (*BnaC.FAD2.b*) and C5 (*BnaC.FAD2.a*) and *BnaSAD3* has six copies, located on A1 (*BnaA.SAD3.a*), C1 (*BnaC.SAD3.a*), A3 (*BnaA.SAD3.b*), C3 (*BnaC.SAD3.b*) and A5 (*BnaA.SAD3.c*) and C5 (*BnaC.SAD3.c*). Mapping results were validated by converting markers to conventional GoldenGate assays. In addition, the methodology was also adapted to identify lesions in target gene families across an EMS-treated population. These targets comprised a specific amplicon of *BnaC.GL2.b* (an orthologue of Arabidopsis *GLABROUS2*, *GL2*) and mixed products from three co-amplified copies of *Bna.FAD2*.

## Results and discussion

### Barcoding of PCR products

**Figure 1:**
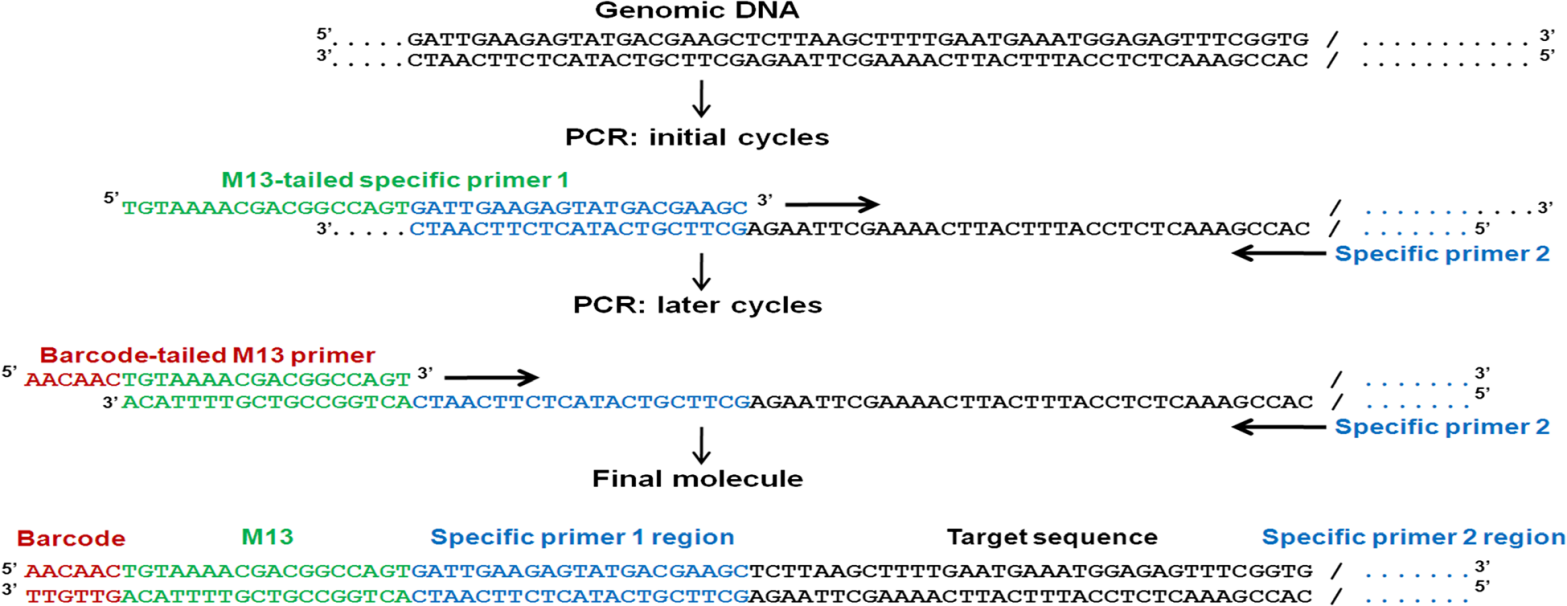
**Schematic showing the addition of the barcode tag during PCR amplification.** In the initial cycles, M13(−21) tails are incorporated into the PCR product however due to the limited amount of specific M13(−21) primer this is replaced with the labelling with the barcode in subsequent cycles.

**Figure 2:**
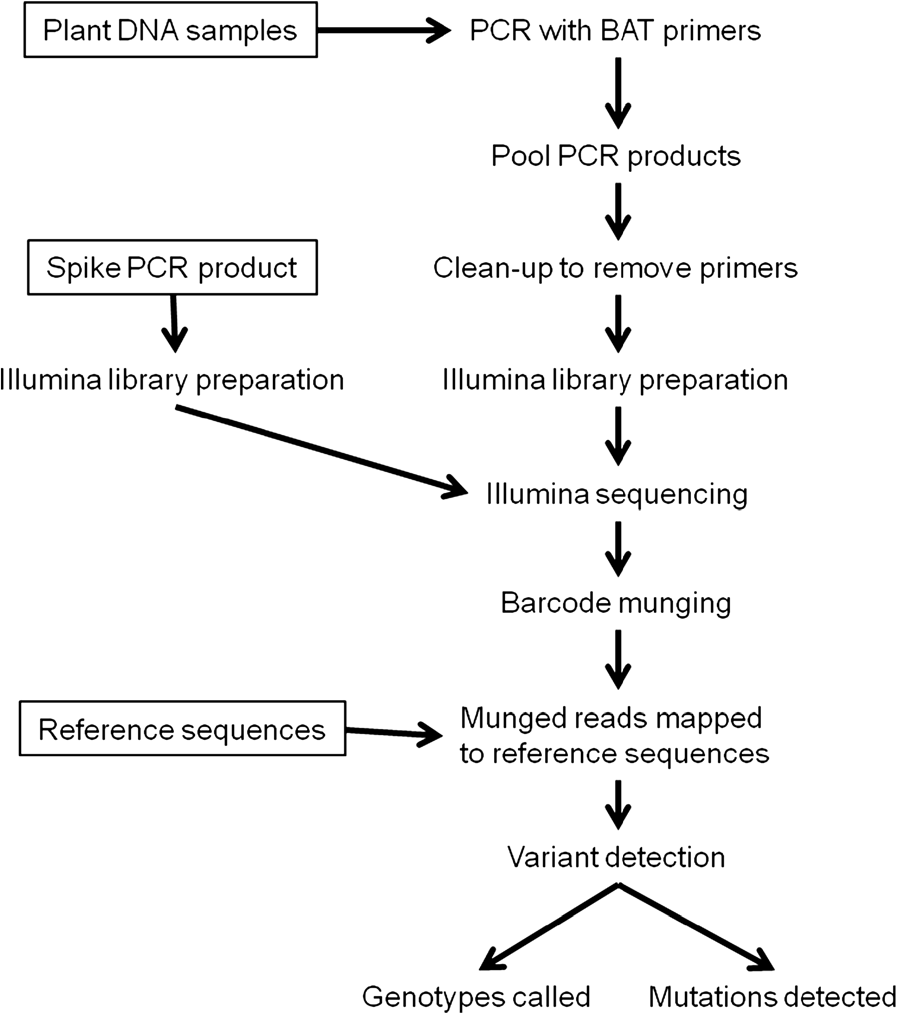
Schematic illustrating the experimental design for the BAT methodology.

An existing method for the addition of fluorescent labels to PCR products [8] was used as the basis for our method for the addition of oligonucleotide barcodes to PCR products. Three oligonucleotides are used in the PCR reactions: (1) a sequence-specific forward primer with M13(−21) tail at its 5’ end, (2) a universal M13(−21) primer with oligonucleotide barcode tail at its 5’ end (hereafter the barcoded amplification tag; BAT) and (3) a sequence-specific reverse primer. By limiting the amount in the reaction of the sequence-specific forward primer with M13(−21) tail, PCR can be conducted in a single-stage reaction in which early rounds of amplification incorporate the universal forward primer and later rounds incorporate the BAT primer. The barcoding process is shown schematically in Figure 1, with the BAT experimental design illustrated schematically in Figure 2.

Three hundred and twenty eight barcodes were tested for amplification and 192, shown in Additional file 1, which produced single, clean bands when amplified with seven test amplicons, were selected for further experimentation.

### SNP genotyping

The use of BAT tailing for multiplexed genotyping was investigated by designing PCR amplicons that contain hemi-SNP markers, i.e. loci that are amplified from both genomes of *B. napus*, only one of which shows allelic variation between cultivar accessions, which are the most abundant type of marker in polyploids [16]. In all, 35 amplicons representing transcribed regions of genes, shown in Additional file 2, were designed to contain hemi-SNP polymorphisms detected previously [16]. Of these, 24 were shown by capillary sequencing of PCR products to contain the expected polymorphism (and some contained additional SNPs), but polymorphisms could were not validated in the remaining 11, which were assumed to be monomorphic. Nevertheless, all 35 were used for the evaluation in order to increase the sequence complexity to more realistic levels. PCR amplification of all 35 amplicons was successfully conducted across a subset of 46 lines of the *B. napus* TNDH mapping population (plus parent cultivars, Tapidor and Ningyou 7) with PCR products amplified in many separate tubes (with different barcodes) and combined for the production of an Illumina sequencing library. The risk of the incorporation of the same base in a large proportion of clusters during one cycle of the Illumina run triggering an error and terminating the run was prevented by the addition of a “spike” sequencing library designed to complement the base composition over the barcode and M13(−21) region of the read. The spike (of ~ 280 base lengths) was added to the sample libraries at an approximately 1:10 ratio of final library molar concentration prior to cluster generation.

One lane (76 base single-end reads) of Illumina sequencing on a GAII platform produced 8,002,602 single-end reads for genotyping. These were analysed for composition, with the results shown in Table 1. Of the sequence reads, 1.1% (89,517) corresponded to the ‘spike’ library and 22% (1,725,988) included the M13(−21) sequence. Of the latter, only 2.3% carried an intact 6 base barcode, 18.4% carried a 5 base barcode, 42.2% carried a 4 base barcode and 37% had barcodes of less than 4 bases. This truncation of the barcodes is likely to have been due to exonucleases degrading the ends of molecules prior to ligation to Illumina adapters. However, as only the final four bases of the barcode were needed for the address space used in the experiment, 63% of the reads carrying M13(−21) tags were usable for genotyping. The sequences truncated to 4 or 5 bases were repaired (computationally) by the addition of 2 bases or 1 base (as appropriate) to restore the full 6 base barcode.

**Table 1:** Distribution of BAT SNP genotyping reads

	**Reads**
**Total**	8,002,602
**Spike**	89,517
**Unmatched**	6,106,250
**M13 detected**	1,725,988
**6 bp BAT**	40,277
**5 bp BAT**	317,373
**4 bp BAT**	729,019
**<4 bp BAT**	639,319
**Munged**	1,086,669
**Called**	699,449

We developed a computational method to allow the robust detection of sequence variants, based on previously-reported methodology involving the mapping of Illumina reads to reference sequences [16,17]. Sequence reads having complete/repaired 6-base barcodes were processed by having their barcodes replicated four-fold by a process termed “munging”. This munging ensures specific mapping of reads to the correct reference sequence, as four mis-matches (the minimum difference between munged barcodes) abolishes the mapping of reads. The munged reads were then mapped using Maq v0.7.1 [27] to reference sequences comprising the specific amplified sequences plus M13(−21) and four-fold munged barcodes of the occupied address space. In this way 64.4% (699,449) of reads processed by Maq were mapped to a reference sequence and associated with individual lines via the munged BAT. A Perl script was used to identify variants in amplified sequences relative to the ancestral reference sequence and call genotypes based on counts of the bases read at the SNP position (subject to a minimum of 20 reads). Conventional marker scoring strings were produced (based on the convention of A allele for that of the maternal parent of the population, Tapidor, B allele for the paternal parent, Ningyou7, and U where the allele could not be called, for example because of insufficient read depth).

**Figure 3:**
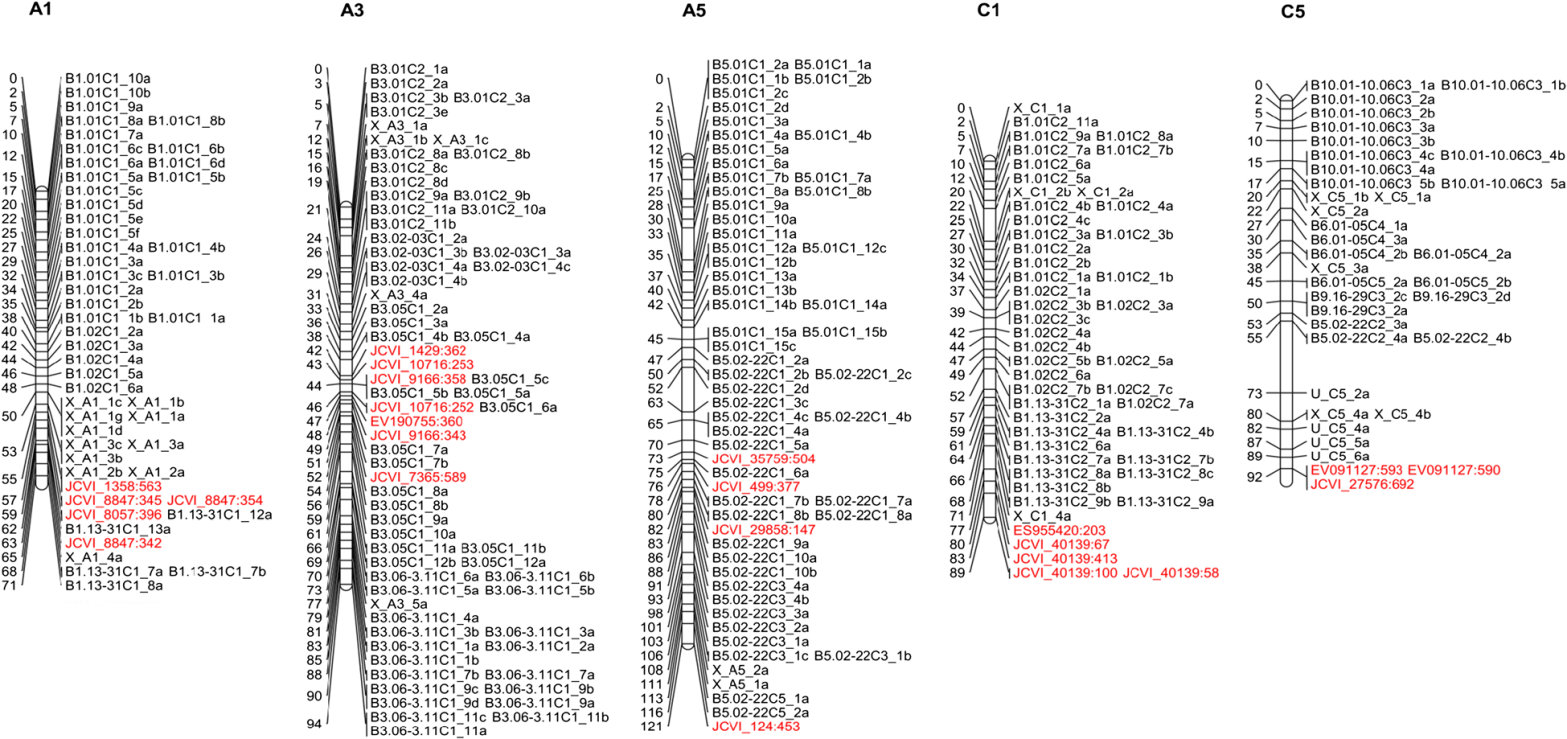
**Linkage groups incorporating newly-developed transcriptome SNP markers.** Transcriptome SNP markers assayed by the BAT method mapped to the anticipated linkage groups **(A1, A5, C1, C5 and C3)** showing and are shown in red.

The resulting scoring strings for 24 markers (derived from 17 amplicons) were successfully linkage mapped, as shown in Figure 3 and listed in Additional file 3, to the expected regions of the genome, i.e. 14 markers on linkage groups A3, A5, C1 and C5, and 10 markers on linkage groups A1, A3 and A5. The remaining SNPs could not be mapped due to lack of polymorphism or because read depth was too low for robust allele-calling. For validation, Illumina GoldenGate platform [11] markers were developed for eight of the unigenes containing SNPs mapped by the BAT method and scored across the population. Also, the scoring strings from the published oilseed rape SNP map [17] for these eight unigenes were retrieved, and the scoring strings for both types of markers compared with those obtained by the BAT method, as shown in Additional file 4. The BAT markers showed a high accuracy of scoring, with only 5 mis-calls in the 483 alleles scored (1.0%).

### Mutation detection

The use of BAT tailing for mutation detection was investigated by searching for EMS-induced mutations in both a locus-specific 176 bp amplicon (from an orthologue of *GL2*; *BnaC.GL2.b*) and a 276 bp amplicon representing three co-amplified loci (from orthologues of *FAD2; BnaA.FAD2.a*, *BnaA.FAD2.b* and *BnaC.FAD2.a*) in the EMS mutagenised population JBnCAB_E (derived from *B. napus* variety Cabriolet). For each of three separate sets, each of which were pooled for the construction of an Illumina sequencing library, 192 different BATs were used for PCR amplification to screen 1,536 lines of the population combined by one-dimensional 8-fold pooling. Three Illumina GAII sequencing lanes (76 base, paired-end reads) were used to screen 4,608 lines for mutations in the amplicons. An average of 56,898,937 reads was obtained per lane. A summary of the analysis of reads and mapping is shown in Table 2. Spike sequences, again added at 1:10 ratio of final library molar concentrations as described for SNP genotyping, accounted for an average of 4.9% (2,791,863) of the reads. M13(−21) sequences were detected in 30.5% (17,329,509) of the reads. Exonuclease activity was greatly reduced compared to the initial experiment, following protocol improvements, with an average of 89.5% (15,505,456) containing an intact barcode, 3.7% (638,532) carrying a 5 bp code and 1.7% (290,934) carrying a 4 bp code. These were repaired where possible. Only 3.8% had barcodes of less than 4 bp and so could not be repaired to allow line identification. As sequence end pairs can be identified, the M13 (−21) sequence an adjacent barcode was computationally added to the paired end (originally corresponding to the sequence-specific reverse primer), resulting in an average of 56.8% (32,317,892) being used for read mapping and mutation detection. Mapping to the amplicon reference sequences resulted in an average of 62.5% (20,195,287) of the 32,317,892 ‘munged’ reads being successfully mapped, a similar proportion to the mapped reads in the SNP genotyping analysis. However, reads were not distributed evenly across amplicons, with the *FAD2* orthologues *BnaA.FAD2.a*, *BnaA.FAD2.b* and *BnaC.FAD2.a* represented by averages of 8.5%, 32% and 4.1% of the reads, respectively. This may have been due to biases in PCR efficiency across the co-amplified loci. Cluster generation from libraries was performed with a 3:1 molar ratio of *BnaFAD2* library to *BnaGL2* library of the two amplicons. However, an average of 55.5% (11,202,018) of the mapped reads was from the *BnaC.GL2.b* amplicon.

**Table 2:** **Numerical breakdown of read distribution on the three Illumina lanes for BAT mutation detection in all *FAD2 *orthologues and *BnaC.GL2.b***

**Reads**	**4**	**5**	**6**	**Average**
**Total**	62,071,296	38,839,694	69,785,822	56,898,937
**Spike**	3,128,126	1,554,316	3,693,148	2,791,863
**Unmatched**	23,262,622	12,912,128	22,169,416	19,448,055
**M13 detected**	17,840,274	12,186,625	21,961,629	17,329,509
**6 bp BAT**	15,859,982	11,258,110	19,398,277	15,505,456
**5 bp BAT**	591,667	338,953	984,976	638,532
**4 bp BAT**	304,755	127,279	440,769	290,934
**<4 bp BAT**	788,808	302,764	891,331	660,968
**Munged**	32,925,286	23,213,290	40,815,100	32,317,892
**Mapped *BnaA.FAD2.a***	1,566,912	1,420,311	2,143,813	1,710,345
**Mapped *BnaA.FAD2.b***	6,579,001	4,804,826	8,002,197	6,462,008
**Mapped *BnaC.FAD2.a***	791,572	644,265	1,026,912	820,916
**Mapped *BnaC.GL2.b***	12,302,580	6,167,201	15,136,272	11,202,018

Following read mapping, Maq was used to identify variants in amplified sequences relative to the wild-type reference sequences, with mutations called on the basis of a number of parameters. An appropriate set of criteria for calling a mutation in a particular pool of eight lines was found to be: (1) at least 10 reads carrying the sequence variant, (2) at least 1% of the calls showing the variant for the mixed amplicon target (i.e. *FAD2*); at least 0.2% for the locus-specific target (i.e. *GL2*), (3) the main variant from the reference base being at least 10-fold more abundant than any other variant bases called at that position, (4) the position should not correspond to that of an inter-paralogue (or inter-homoeologue) polymorphism. Under these criteria, we detected 14, 19, 8 and 10 lines carrying mutations in *BnaA.FAD2.a*, *BnaA.FAD2.b*, *BnaC.FAD2.a* and *BnaC.GL2.b*, respectively. A subset of these identified mutations was tested by PCR amplification from the original (non-pooled) DNA samples using locus-specific primers. All of the 2, 4, 2 and 7 tested putative mutations in *BnaA.FAD2.a*, *BnaA.FAD2.b*, *BnaC.FAD2.a* and *BnaC.GL2.b*, respectively, were confirmed to be present, as shown in Table 3. Other mutations could have been present in the population but escaped detection as a result of too few sequence reads for some lines; a consequence of imbalances in the amounts of DNA pooled per line for PCR amplification and/or that pooled for library construction, the latter of which was not normalised in this experiment.

**Table 3:** **List of mutations detected within the three Brassica orthologues of *FAD2 *and *BnaC.GL2.b***

**Lane**	**BAT**	**Gene**	**Amplicon position**	**Base**	**Reads**				**Ratio**	**Wild type AA**	**Mutant AA**
					**A**	**C**	**G**	**T**			
6	B00146	*BnaC.FAD2.a*	37	G	183	0	1640	0	0.111585		Glu	Lys
5	B00282	*BnaC.FAD2.a*	45	C	0	3555	0	246	0.069198		Leu	Leu
4	B00009	*BnaC.FAD2.a*	46	C	7	5431	2	77	0.014178		Glu	STOP
4	B00282	*BnaC.FAD2.a*	57	C	0	2032	1	164	0.080709		Ile	Ile
4	B00136	*BnaC.FAD2.a*	59	C	0	2176	0	59	0.027114		Ser	Phe
4	B00077	*BnaC.FAD2.a*	68	G	137	0	1524	1	0.089895		Gly	Asp
6	B00087	*BnaC.FAD2.a*	227	C	0	143	0	14	0.097902		Ser	Leu
6	B00040	*BnaC.FAD2.a*	249	G	11	0	931	0	0.011815		Arg	Arg
6	B00207	*BnaA.FAD2.a*	43	C	3	14174	0	147	0.010371		N/A	N/A
4	B00108	*BnaA.FAD2.a*	45	C	2	12350	0	319	0.02583		N/A	N/A
5	B00136	*BnaA.FAD2.a*	45	C	0	4566	0	298	0.065265		N/A	N/A
6	B00041	*BnaA.FAD2.a*	45	C	1	5883	0	68	0.011559		N/A	N/A
4	B00208	*BnaA.FAD2.a*	48	G	309	0	5574	0	0.055436		N/A	N/A
5	B00119	*BnaA.FAD2.a*	48	G	940	8	15594	0	0.06028		N/A	N/A
6	B00114	*BnaA.FAD2.a*	63	C	1	8285	0	88	0.010622		N/A	N/A
6	B00147	*BnaA.FAD2.a*	63	C	2	9325	0	861	0.092332		N/A	N/A
4	B00108	*BnaA.FAD2.a*	64	G	1065	3	10533	0	0.101111		N/A	N/A
6	B00134	*BnaA.FAD2.a*	69	C	4	8271	0	237	0.028654		N/A	N/A
4	B00058	*BnaA.FAD2.a*	225	C	2	5339	0	316	0.059187		N/A	N/A
6	B00075	*BnaA.FAD2.a*	238	G	181	0	1314	1	0.137747		N/A	N/A
4	B00044	*BnaA.FAD2.a*	248	G	40	0	2640	0	0.015152		N/A	N/A
5	B00282	*BnaA.FAD2.a*	248	G	29	0	846	2	0.034279		N/A	N/A
4	B00073	*BnaA.FAD2.b*	37	G	53	0	1062	0	0.049906		N/A	N/A
5	B00285	*BnaA.FAD2.b*	39	G	27	0	1471	0	0.018355		N/A	N/A
5	B00213	*BnaA.FAD2.b*	39	G	6170	36	59711	4	0.103331		N/A	N/A
6	B00128	*BnaA.FAD2.b*	40	C	9	30818	0	1156	0.037511		N/A	N/A
4	B00027	*BnaA.FAD2.b*	43	C	26	72577	1	6652	0.091654		N/A	N/A
4	B00180	*BnaA.FAD2.b*	45	C	9	26921	0	824	0.030608		N/A	N/A
4	B00260	*BnaA.FAD2.b*	45	C	11	31793	2	1069	0.033624		N/A	N/A
5	B00135	*BnaA.FAD2.b*	45	C	1	4675	0	114	0.024385		N/A	N/A
5	B00211	*BnaA.FAD2.b*	45	C	9	67246	3	5206	0.077417		N/A	N/A
5	B00243	*BnaA.FAD2.b*	45	C	5	87630	1	4416	0.050394		N/A	N/A
6	B00299	*BnaA.FAD2.b*	48	G	335	1	13245	1	0.025293		N/A	N/A
4	B00090	*BnaA.FAD2.b*	59	C	34	127784	1	2404	0.018813		N/A	N/A
5	B00087	*BnaA.FAD2.b*	59	C	3	8544	0	744	0.087079		N/A	N/A
5	B00223	*BnaA.FAD2.b*	63	C	28	64330	0	12219	0.189942		N/A	N/A
6	B00250	*BnaA.FAD2.b*	63	C	2	1965	0	413	0.210178		N/A	N/A
5	B00111	*BnaA.FAD2.b*	68	G	249	1	3179	1	0.078327		N/A	N/A
6	B00195	*BnaA.FAD2.b*	219	C	0	2666	0	28	0.010503		N/A	N/A
6	B00118	*BnaA.FAD2.b*	237	G	20	0	2055	0	0.009732		N/A	N/A
5	B00105	*BnaA.FAD2.b*	265	G	97	0	352	0	0.275568		N/A	N/A
6	B00118	*BnaC.GL2.b*	46	C	14	46512	3	1058	0.022747		intron	
5	B00249	*BnaC.GL2.b*	65	G	330	2	20270	9	0.01628		Ile	Ile
4	B00104	*BnaC.GL2.b*	68	C	1	7275	1	67	0.00921		Arg	Arg
4	B00064	*BnaC.GL2.b*	109	C	6	32767	4	121	0.003693		Val	Met
4	B00307	*BnaC.GL2.b*	110	C	7	38693	2	784	0.020262		Ala	Ala
4	B00076	*BnaC.GL2.b*	113	C	5	32262	0	70	0.00217		Lys	Lys
6	B00091	*BnaC.GL2.b*	122	C	11	78003	2	1427	0.018294		Leu	Leu
6	B00228	*BnaC.GL2.b*	130	C	5	16523	2	191	0.01156		Ala	Thr
5	B00147	*BnaC.GL2.b*	143	C	1	14670	4	220	0.014997		Trp	STOP
6	B00115	*BnaC.GL2.b*	143	C	17	76370	43	347	0.004544		Trp	STOP

## Conclusion

We describe a robust and cost-effective methodology for oligonucleotide barcoding via one-step incorporation into PCR products suitable for Illumina sequencing by standard workflows. Computational methods then allow the deconvolution of the sequences and both the scoring of known polymorphisms and the detection of mutations. We have demonstrated the use of 4 base oligonucleotide barcode address space, but using this methodology much larger numbers of PCR products could be pooled and analysed if more of the address space of the tails (which could be longer than 6 bases) were used, although the inclusion of some unused bases helps mitigate the effects of any exonuclease contamination. The panel of 192 BAT oligonucleotides synthesised and validated can be re-used with many different M13-tailed specific primers, making the approach much more cost effective than synthesising locus-specific PCR primers or locus-specific library adapters incorporating barcodes.

Current multiplexing systems on next generation sequencing platforms rely on the barcoding of the library production adapters. Barcoding the product before library construction provides a system of multiplexing with minimal library production costs. As it does not involve modification of adapters used for sequencing library production, pooled PCR products can be used in standard Illumina workflows, including current indexing techniques, so can enable higher-order multiplexing within each lane of the sequencer and (potentially) the analysis of thousands of pooled samples in a single lane, involving the production of only tens of sequencing libraries. Multiple different target loci can also be deconvoluted even when the same BATs have been used (because the majority of the amplicon sequenced will differ between targets).

Our comparison of results between the GoldenGate SNP platform and BAT-tailing has shown the method provides a robust and economical system for SNP marker genotyping, even in polyploid species. It is particularly suitable for applications such as marker-assisted selection within crop breeding programs as modest numbers of markers can be screened across thousands of individuals. Indeed, the technology can be used to target and develop markers for causative sequence changes within genes, which are much preferred (where they can be identified) as they are “perfect” markers, not subject to the breakdown often associated with markers based on polymorphisms that are merely linked genetically. Although the lengths of amplicons that can be sequenced using low-cost platforms such as Illumina are presently shorter than can be assessed for mutations by conventional TILLING (~1 kb), advances in technology continue to increase read lengths with 400 bp now being achievable with the Ion Torrent platform, for example [28]. Already, however, the additional cost of amplifying multiple smaller amplicons for analysis involving BAT-tailing is more than off-set by the efficiency of the process, which is able to detect mutations in multiple co-amplified PCR-products. The ability to use the methodology to detect mutations within mixed amplicons establishes BAT-tailing as a valuable technique for exploiting mutation breeding approaches in polyploids.

## Methods

### Materials and methods

#### BAT PCR amplification

PCR for this barcoded amplified tag strategy based on the fluorolabelled M13 protocol described by Scheulke [8] was performed using 5 μl of 20 ng/μl DNA, 2 μl 10 × PCR buffer (500 mM KCl, 100 mM Tris–HCl (pH 9.0), 1% Triton X-100 15 mM MgCl_2_ (supplied with AmplitaqTaq Gold)), 0.5 μl forward primer (0.5 mM), 2.3 μl reverse primer (2 mM), 2.3 μl barcoded M13(−21) primer (2 mM), 1.3 μl dNTPs (2 μM Invitrogen Cat. No. 10297–018), 0.2 μl Amplitaq Gold 5 u/μl (Applied Biosystems Cat. No. 4311820), 6.4 μl ddH_2_O on the following cycle: 94°C for 5 minutes, 40 × (94°C for 30 seconds, 49°C for 30 seconds, 72°C for 30 seconds), 72°C for 7 minutes, store at 8°C.

#### SNP marker assay design and library production

SNP markers for testing were designed to assay hemi-SNP polymorphisms in unigenes representing orthologues of gene models in the *A. thaliana* genome that are close to genes involved in lipid biosynthesis (*FAD2*, At3g12120; *SAD3* tandem triplicate, At3g02610/20/30). Using BLAST alignment of *Brassica* unigenes against *A. thaliana* CDS models, putative exons were identified (based on the assumption that introns will generally be in the same place in orthologues in related species such as *A. thaliana* and *B. napus*). PCR primers were designed within these putative exon sequences with the aim of designing amplicons of ~130 bases or ~280 bases. PCR products were validated for the expected polymorphism by amplification from cultivars Tapidor and Ningyou 7, capillary sequencing and inspection of trace files. Reference sequences for the mapping of Illumina sequence reads were designed to represent the amplified sequences with the ancestral base at the hemi-SNP position (i.e. the resolved base allele) (see Additional file 2, for reference sequences).

Each of the 46 TNDH lines to be genotyped (plus Tapidor and Ningyou7) was assigned a specific barcode (see Additional file 5 for barcode allocations). PCR products were pooled for each of the 35 assays, PAGE was used to clean and concentrate the sample prior to Illumina mRNA-seq single-end library production from the adapter ligation reaction stage of the protocol following the manufacturer’s instructions. Library preparation methods were as described in Bancroft *et al.* 2011 [17].

#### Spike sample preparation

Ordinarily the Illumina real time analysis software would fault a situation in which all clusters report incorporation of the same nucleotide on a given cycle, yet this would occur for the universal primer portion of reads derived from the PCR amplicons. To overcome this, experimental samples were spiked with a 1:10 ratio, based on library molar concentration (libraries being nomalised to approximately 10 nM), with DNA comprising sequences that should complement those of the M13 and barcode regions. Two sets of spike samples were prepared, one of ~130 bases in length, the other of ~280 bases on length, each comprising two different amplicons. For the ~130 base spike, PCR was conducted using DNA from *B. napus* cultivar Tapidor and primer pairs R00005 and D00002 (BBNNNNVHVBBBBDHBDHHDDBHVCAGAGCACGTGGAGCAG and TGCATTTTTCTGTTGGTTTTTCACC, respectively) and R00006 and D00006 (BBNNNNVHVBBBBDHBDHHDDBHVCGTACATTTGAGAGACGTTCC and GAGCAGCGAGCAATGCAGC, respectively), with the amplification products being pooled. For the ~280 base spike, PCR was conducted using DNA from *B. napus* cultivar Tapidor and primer pairs R00008 and D00032 (BBNNNNVHVBBBBDHBDHHDDBHVTGACGGTGAAGTACCTCCTC and GATCTCCGCTCCAAGATGG, respectively) and R00009 and D00041 (BBNNNNVHVBBBBDHBDHHDDBHVGATGTCTTCAAGAGCCCAGC and GATAGCAGTGACAATATCTCC, respectively), with the amplification products being pooled. Libraries for these spikes were produced following the same protocol as for the pooled barcoded experimental samples, resulting in one library for spiking the sequencing of smaller size range amplicons (~130 base spike) and one library for spiking the sequencing of larger size range amplicons (~280 base spike). Quality checking and processing were conducted as previously described [17].

#### Illumina sequencing

Illumina sequencing on the GAII platform, quality checking and processing were conducted as described previously [17]. Illumina base-calling files were processed using GERALD to produce paired sequence files containing 80 base reads for each sample in FASTQ format. The Illumina FASTQ format was converted to Sanger FASTQ format before further processing.

#### Data processing

To address reads back to barcodes, and hence individual PCR wells or pools, we developed a novel method based on techniques reported previously [16,17]. We used Maq v0.7.1 [27] to align reads against reference sequences comprising the target amplicons and to identify mismatches (SNPs or mutations). However, mismatches between read and reference sequence within the barcode region (that encodes sample origin) must be partitioned from those in the target region (that would signal SNPs or mutations). With very high levels of multiplexing (as in this experiment), in which a large part of the 4 K address space offered by 6 base barcodes is occupied, individual barcodes may differ by only one base. Ordinarily this could result in cross-alignment, i.e. wild type reads derived from a given barcode could potentially be mapped to an incorrect barcode closely neighbouring in address space. We countered this problem by replacing one-to-one, *in silico*, the real six base barcodes with an artificial construct derived from a bigger address space and thus able to carry more discriminating information. Our simple implementation was to replicate the barcode four-fold (“munging”) such that a one base difference became amplified to four, thus defeating cross-alignment by Maq with default parameters. This was done with simple Perl scripts mung_barcoded_reads.pl (Additional file 6) and mung_refseqs.pl (Additional file 7) operating on the reads and the references respectively. During our analysis it was found that a proportion of reads had incomplete barcodes which could nevertheless be rescued and so only those with a perfect match to the M13 primer and at least four bases of the barcode (and which could be reconstructed back to a unique, full barcode) were used.

Reads corresponding to the PCR spike amplicons were identified and removed and individual lines were associated with their sequence from the BAT. The munged reads were aligned by Maq, with default parameters, to the reference sequence for each marker and the genotype called, based on counts of the bases read at the SNP position, but only if the SNP position was covered by at least 20 high quality reads (otherwise the allele is defined as unclassified, U) using Perl script BAT_SNPs.pl (Additional file 8).

#### SNP marker mapping

Linkage mapping was performed using Joinmap version 3.0 [29][30] on data from 42 of the 46 lines of the TNDH population sequenced (the remaining 4 lines produced relative few mapped sequences, hence a large proportion of alleles remained unclassified). BAT SNP markers were added to the latest version of an existing SNP map based on this population [17], with the previously mapped markers assigned to a fixed order. Mapping criteria were as follows: with the default recombination (REC) threshold (0.4) and logarithm of the odds of the differences (LOD) value (2.0) [30]. The mean chi squared contributions, the markers average contribution to the goodness-of-fit, were less than 2.

#### Mutation screen amplicon design and DNA pooling strategy

Primers were designed to produce a 276 bp mixed amplicon of the three *BnaFAD2* copies present within *B. napus* cultivar Cabriolet and a 176 bp amplicon specific for *BnaC.GL2.b*. (see Additional file 9) The forward primer of each amplicon had a 5’ M13(−21) tail (Additional file 5: Table S5).

DNA concentrations were determined for the *B. napus* Cabriolet EMS population, JBnCAB_E, using PicoGreen (Molecular Probes, Invitrogen Corporation, Carlsbad, California, USA) against a universal DNA concentration standard on a Tecan Genios plate reader, DNA from 4608 0.6 and 0.8% EMS lines was normalised to1 ng per ul. DNA was then pooled using a one-dimensional eight-fold pooling strategy.

192 separate M13 primers labelled with a unique six bp ‘barcode’, the sequence of which could be used to identify the pool from which a product had originated,were used to allow two pooled plates, each well of which had a uniquely tagged product, to be combined and run per Illumina lane. Different target genes amplified and tagged with the same barcodes could be combined and run within the same lane.

#### Sequencing library construction

Handling the two amplicons separately, PCR product from each pair of 96-well plates labelled with 192 BAT sequences addressed to individual wells was pooled and 30 ul run through Min-Elute reaction columns (Qiagen cat. No. 28204) to produce 3 × 10 ul cleaned, concentrated product. 2 ul of elute from each column was run on the Agilent DNA 1000 Bioanalyzer to check quality and to quantify the DNA concentration before the remaining elute was combined. Concentration was adjusted to 20-25 ng/ul and 23 ul used for Illumina mRNA-seq paired-end library production from the adapter ligation reaction stage of the protocol following the manufacturer’s instructions. Libraries were normalised to ~10 nM and a 3:1 molar ratio combined from a single library from each amplicon before clustering. Spike library was added as for the SNP marker assay.

#### Mutation data processing

Mutations were detected using an adaption of the program used for SNP assay. The munged reads were competitively aligned by Maq, with default parameters, to the munged references. The maq pileup –v command was then used to generate text files encapsulating these alignments and associated base quality information. A Perl script TILLING_parse.pl (Additional file 10) was developed to extract the called base frequency compositions at each EMS-mutable base within the target amplicon, using base qualities, by accessing pre-indexed versions of the pileup files.

Pools were identified as potentially containing mutant line(s) based on a number of heuristics. First, the absolute number of reads carrying an EMS mutation candidate was required to exceed 10, then the signal, the ratio of calls of this candidate to the reference (wild-type) base, was required to be greater than 0.002 for specific amplicons (based on expectation from the depth of multiplexing), 0.01 for product from mixed homologue amplicons, and finally the signal-to-noise ratio (calculated as the ratio of calls of the candidate to calls of the other two possible base changes) was required to be greater than 10-fold.

#### Confirmation of mutations

PCR of specific amplicons identified to contain candidate mutations was performed on the individuals from positively identified pools using the original BnaC.GL2.b primers and the *BnaFAD2* primers and combinations, detailed in Additional files 11, 12 and 13, on a standard 57°C PCR cycle before preparation, sequencing and mutation detection as described previously.

## Competing interests

The authors declare no competing financial interests.

## Authors’ contributions

IB and RW conceived and planned the project. RW, FF, ES and LC carried out the experiments. IB, RW, MT, FF and CLM performed data analysis. JP developed and conducted GoldenGate assays and validated markers, IB and RW wrote the manuscript and all authors reviewed it. All authors read and approved the final manuscript.

## Supplementary Material

Additional file 1 **The 192 barcoded M13 primers for BAT of amplicons identified to produce clean, single band PCR products.** Word table of barcoded primer details. Click here for file

Additional file 2 **Transcriptome SNP markers assayed using BAT.** Word table of primer sequences, SNP details and unigene positions for assayed markers. Click here for file

Additional file 3 **Linkage mapping of SNP markers scored by BAT method.** Word table of mapping positions of SNP markers from genotyping determined by GoldenGate and BAT scoring and read depth details for BAT SNP scoring. Click here for file

Additional file 4 **Comparison of allele scores generated on different platforms.** MS Excel file containing allele scoring strings for SNP markers. Click here for file

Additional file 5 Table S5 Barcode assignment for SNP transcriptome marker genotyping using BAT. Word table containing individual population line barcode assignments. Click here for file

Additional file 6 Perl script to endoreduplicate the 6nt barcodes in tagged, multiplexed Illumina reads. Click here for file

Additional file 7 Perl script to attach 6nt barcodes to amplicons and endoreduplicate to create pseudoreference sequences. Click here for file

Additional file 8 Perl script for SNP genotype scoring from Illumina reads. Click here for file

Additional file 9 **BAT mutation detection amplicons.** Amplified sequences are shown for *Bna.FAD2* family (panel a) and *BnaC.GL2.b* (panel b). Regions of the amplicons are colour-coded: barcode (blue), M13 (red) and specific primer (green) positions. Regions of the amplicons that can be re-sequenced with 76 base reads, as used in the experiments, are not coloured. Regions not covered by the 76 bp read are coloured grey. Description: Illustration of sequence regions covered within the BAT mutation screen. Click here for file

Additional file 10 Perl script to parse Maq pileup file from alignment of Illumina reads from EMS population vs reference sequences. Click here for file

Additional file 11 ***Bna.FAD2* coamplification and *BnaC.GL2.b-*specific primers used to produce the amplicons for BAT mutation screening.** Word table containing primer details of screened amplicons. Click here for file

Additional file 12 **Primers used for the amplification of *Bna.FAD2* loci.** Word table containing primers used for the amplification of *Bna.FAD2.* Click here for file

Additional file 13 **Primer combinations required for the locus-specific amplification of *FAD2* orthologues.** Word table containing primer combinations. Click here for file
