# Supplementary material for: Sequencing-based variant detection in the polyploid crop oilseed rape
Source: BMC Plant Biol. 2013 Aug 6;13:111. doi: 10.1186/1471-2229-13-111 (PMC3750413; doi:10.1186/1471-2229-13-111)
Supplement: Additional file 1 — The 192 barcoded M13 primers for BAT of amplicons identified to produce clean, single band PCR products. Word table of barcoded primer details. [file 1471-2229-13-111-S1.docx]

| **Barcode Primer** | **Sequence (5'-3')** | **Barcode Primer** | **Sequence (5'-3')** |
| --- | --- | --- | --- |
|  |  |  |  |
| **B00002** | AACAAGTGTAAAACGACGGCCAGT | **B00071** | AAGTGGTGTAAAACGACGGCCAGT |
| **B00003** | AACACATGTAAAACGACGGCCAGT | **B00073** | AATAAGTGTAAAACGACGGCCAGT |
| **B00004** | AACACCTGTAAAACGACGGCCAGT | **B00074** | AATACATGTAAAACGACGGCCAGT |
| **B00005** | AACACGTGTAAAACGACGGCCAGT | **B00075** | AATACCTGTAAAACGACGGCCAGT |
| **B00006** | AACAGATGTAAAACGACGGCCAGT | **B00076** | AATACGTGTAAAACGACGGCCAGT |
| **B00007** | AACAGCTGTAAAACGACGGCCAGT | **B00077** | AATAGATGTAAAACGACGGCCAGT |
| **B00008** | AACAGGTGTAAAACGACGGCCAGT | **B00078** | AATAGCTGTAAAACGACGGCCAGT |
| **B00009** | AACATATGTAAAACGACGGCCAGT | **B00079** | AATAGGTGTAAAACGACGGCCAGT |
| **B00010** | AACATCTGTAAAACGACGGCCAGT | **B00080** | AATCAATGTAAAACGACGGCCAGT |
| **B00011** | AACATGTGTAAAACGACGGCCAGT | **B00081** | AATCACTGTAAAACGACGGCCAGT |
| **B00012** | AACCAATGTAAAACGACGGCCAGT | **B00085** | AATCGATGTAAAACGACGGCCAGT |
| **B00013** | AACCACTGTAAAACGACGGCCAGT | **B00087** | AATCGGTGTAAAACGACGGCCAGT |
| **B00014** | AACCAGTGTAAAACGACGGCCAGT | **B00088** | AATGAATGTAAAACGACGGCCAGT |
| **B00015** | AACCGATGTAAAACGACGGCCAGT | **B00090** | AATGAGTGTAAAACGACGGCCAGT |
| **B00016** | AACCGCTGTAAAACGACGGCCAGT | **B00091** | AATGCATGTAAAACGACGGCCAGT |
| **B00017** | AACCGGTGTAAAACGACGGCCAGT | **B00092** | AATGCCTGTAAAACGACGGCCAGT |
| **B00018** | AACCTATGTAAAACGACGGCCAGT | **B00094** | AATGGATGTAAAACGACGGCCAGT |
| **B00019** | AACCTCTGTAAAACGACGGCCAGT | **B00097** | ACCAACTGTAAAACGACGGCCAGT |
| **B00020** | AACCTGTGTAAAACGACGGCCAGT | **B00103** | ACCAGCTGTAAAACGACGGCCAGT |
| **B00022** | AACGACTGTAAAACGACGGCCAGT | **B00104** | ACCAGGTGTAAAACGACGGCCAGT |
| **B00023** | AACGAGTGTAAAACGACGGCCAGT | **B00105** | ACCATATGTAAAACGACGGCCAGT |
| **B00027** | AACGGATGTAAAACGACGGCCAGT | **B00108** | ACACAATGTAAAACGACGGCCAGT |
| **B00029** | AACGTATGTAAAACGACGGCCAGT | **B00109** | ACACACTGTAAAACGACGGCCAGT |
| **B00030** | AACGTCTGTAAAACGACGGCCAGT | **B00110** | ACACAGTGTAAAACGACGGCCAGT |
| **B00031** | AACGTGTGTAAAACGACGGCCAGT | **B00111** | ACACGATGTAAAACGACGGCCAGT |
| **B00033** | AACTACTGTAAAACGACGGCCAGT | **B00112** | ACACGCTGTAAAACGACGGCCAGT |
| **B00034** | AACTAGTGTAAAACGACGGCCAGT | **B00113** | ACACGGTGTAAAACGACGGCCAGT |
| **B00035** | AACTCATGTAAAACGACGGCCAGT | **B00114** | ACACTATGTAAAACGACGGCCAGT |
| **B00036** | AACTCCTGTAAAACGACGGCCAGT | **B00115** | ACACTCTGTAAAACGACGGCCAGT |
| **B00037** | AACTCGTGTAAAACGACGGCCAGT | **B00116** | ACACTGTGTAAAACGACGGCCAGT |
| **B00040** | AACTGGTGTAAAACGACGGCCAGT | **B00117** | ACCGAATGTAAAACGACGGCCAGT |
| **B00041** | AAGAACTGTAAAACGACGGCCAGT | **B00118** | ACCGACTGTAAAACGACGGCCAGT |
| **B00042** | AAGAAGTGTAAAACGACGGCCAGT | **B00119** | ACCGAGTGTAAAACGACGGCCAGT |
| **B00043** | AAGACATGTAAAACGACGGCCAGT | **B00120** | ACCGCATGTAAAACGACGGCCAGT |
| **B00044** | AAGACCTGTAAAACGACGGCCAGT | **B00125** | ACCGTATGTAAAACGACGGCCAGT |
| **B00045** | AAGACGTGTAAAACGACGGCCAGT | **B00126** | ACCGTCTGTAAAACGACGGCCAGT |
| **B00046** | AAGAGATGTAAAACGACGGCCAGT | **B00128** | ACCTAATGTAAAACGACGGCCAGT |
| **B00047** | AAGAGCTGTAAAACGACGGCCAGT | **B00129** | ACCTACTGTAAAACGACGGCCAGT |
| **B00057** | AAGGAATGTAAAACGACGGCCAGT | **B00130** | ACCTAGTGTAAAACGACGGCCAGT |
| **B00058** | AAGGACTGTAAAACGACGGCCAGT | **B00134** | ACCTGATGTAAAACGACGGCCAGT |
| **B00059** | AAGGAGTGTAAAACGACGGCCAGT | **B00135** | ACCTGCTGTAAAACGACGGCCAGT |
| **B00063** | AAGTAATGTAAAACGACGGCCAGT | **B00136** | ACCTGGTGTAAAACGACGGCCAGT |
| **B00064** | AAGTACTGTAAAACGACGGCCAGT | **B00137** | ACGAACTGTAAAACGACGGCCAGT |
| **B00065** | AAGTAGTGTAAAACGACGGCCAGT | **B00139** | ACGACATGTAAAACGACGGCCAGT |
| **B00066** | AAGTCATGTAAAACGACGGCCAGT | **B00141** | ACGACGTGTAAAACGACGGCCAGT |
| **B00067** | AAGTCCTGTAAAACGACGGCCAGT | **B00145** | ACGCAATGTAAAACGACGGCCAGT |
| **B00068** | AAGTCGTGTAAAACGACGGCCAGT | **B00146** | ACGCACTGTAAAACGACGGCCAGT |
| **B00069** | AAGTGATGTAAAACGACGGCCAGT | **B00147** | ACGCAGTGTAAAACGACGGCCAGT |
| **B00070** | AAGTGCTGTAAAACGACGGCCAGT | **B00148** | ACGCCATGTAAAACGACGGCCAGT |
|  |  |  |  |

| **Barcode Primer** | **Sequence (5'-3')** | **Barcode Primer** | **Sequence (5'-3')** |
| --- | --- | --- | --- |
|  |  |  |  |
| **B00150** | ACGCGATGTAAAACGACGGCCAGT | **B00241** | AGGCAATGTAAAACGACGGCCAGT |
| **B00154** | ACGGACTGTAAAACGACGGCCAGT | **B00242** | AGGCACTGTAAAACGACGGCCAGT |
| **B00155** | ACGGAGTGTAAAACGACGGCCAGT | **B00243** | AGGCAGTGTAAAACGACGGCCAGT |
| **B00163** | ACGTCCTGTAAAACGACGGCCAGT | **B00244** | AGGCCATGTAAAACGACGGCCAGT |
| **B00165** | ACGTGATGTAAAACGACGGCCAGT | **B00246** | AGGCGATGTAAAACGACGGCCAGT |
| **B00166** | ACGTGCTGTAAAACGACGGCCAGT | **B00249** | AGAGAATGTAAAACGACGGCCAGT |
| **B00174** | ACTAGCTGTAAAACGACGGCCAGT | **B00250** | AGAGACTGTAAAACGACGGCCAGT |
| **B00175** | ACTAGGTGTAAAACGACGGCCAGT | **B00251** | AGAGAGTGTAAAACGACGGCCAGT |
| **B00178** | ACTCAGTGTAAAACGACGGCCAGT | **B00252** | AGAGCATGTAAAACGACGGCCAGT |
| **B00179** | ACTCCATGTAAAACGACGGCCAGT | **B00253** | AGAGCCTGTAAAACGACGGCCAGT |
| **B00180** | ACTCCGTGTAAAACGACGGCCAGT | **B00254** | AGAGCGTGTAAAACGACGGCCAGT |
| **B00181** | ACTCGATGTAAAACGACGGCCAGT | **B00255** | AGGTAATGTAAAACGACGGCCAGT |
| **B00185** | ACTGACTGTAAAACGACGGCCAGT | **B00257** | AGGTAGTGTAAAACGACGGCCAGT |
| **B00189** | ACTGCGTGTAAAACGACGGCCAGT | **B00258** | AGGTCATGTAAAACGACGGCCAGT |
| **B00190** | ACTGGATGTAAAACGACGGCCAGT | **B00260** | AGGTCGTGTAAAACGACGGCCAGT |
| **B00193** | AGCAACTGTAAAACGACGGCCAGT | **B00263** | AGGTGGTGTAAAACGACGGCCAGT |
| **B00194** | AGCAAGTGTAAAACGACGGCCAGT | **B00264** | AGTAACTGTAAAACGACGGCCAGT |
| **B00195** | AGCACATGTAAAACGACGGCCAGT | **B00265** | AGTAAGTGTAAAACGACGGCCAGT |
| **B00196** | AGCACCTGTAAAACGACGGCCAGT | **B00266** | AGTACATGTAAAACGACGGCCAGT |
| **B00197** | AGCACGTGTAAAACGACGGCCAGT | **B00267** | AGTACCTGTAAAACGACGGCCAGT |
| **B00198** | AGCAGATGTAAAACGACGGCCAGT | **B00268** | AGTACGTGTAAAACGACGGCCAGT |
| **B00202** | AGCATCTGTAAAACGACGGCCAGT | **B00269** | AGTAGATGTAAAACGACGGCCAGT |
| **B00203** | AGCATGTGTAAAACGACGGCCAGT | **B00272** | AGTCAATGTAAAACGACGGCCAGT |
| **B00204** | AGCCAATGTAAAACGACGGCCAGT | **B00273** | AGTCACTGTAAAACGACGGCCAGT |
| **B00205** | AGCCACTGTAAAACGACGGCCAGT | **B00274** | AGTCAGTGTAAAACGACGGCCAGT |
| **B00206** | AGCCAGTGTAAAACGACGGCCAGT | **B00275** | AGTCCATGTAAAACGACGGCCAGT |
| **B00207** | AGCCGATGTAAAACGACGGCCAGT | **B00276** | AGTCCGTGTAAAACGACGGCCAGT |
| **B00208** | AGCCGCTGTAAAACGACGGCCAGT | **B00279** | AGTCGGTGTAAAACGACGGCCAGT |
| **B00209** | AGCCGGTGTAAAACGACGGCCAGT | **B00280** | AGTGAATGTAAAACGACGGCCAGT |
| **B00211** | AGCCTCTGTAAAACGACGGCCAGT | **B00281** | AGTGACTGTAAAACGACGGCCAGT |
| **B00212** | AGCCTGTGTAAAACGACGGCCAGT | **B00282** | AGTGAGTGTAAAACGACGGCCAGT |
| **B00213** | AGCGAATGTAAAACGACGGCCAGT | **B00283** | AGTGCATGTAAAACGACGGCCAGT |
| **B00216** | AGCGCATGTAAAACGACGGCCAGT | **B00284** | AGTGCCTGTAAAACGACGGCCAGT |
| **B00219** | AGCGGATGTAAAACGACGGCCAGT | **B00285** | AGTGCGTGTAAAACGACGGCCAGT |
| **B00223** | AGCGTGTGTAAAACGACGGCCAGT | **B00293** | AGACGCTGTAAAACGACGGCCAGT |
| **B00224** | AGCTAATGTAAAACGACGGCCAGT | **B00297** | AGACTGTGTAAAACGACGGCCAGT |
| **B00227** | AGCTCATGTAAAACGACGGCCAGT | **B00299** | ATACACTGTAAAACGACGGCCAGT |
| **B00228** | AGCTCCTGTAAAACGACGGCCAGT | **B00300** | ATACAGTGTAAAACGACGGCCAGT |
| **B00229** | AGCTCGTGTAAAACGACGGCCAGT | **B00303** | ATACGGTGTAAAACGACGGCCAGT |
| **B00230** | AGCTGATGTAAAACGACGGCCAGT | **B00305** | ATACTCTGTAAAACGACGGCCAGT |
| **B00231** | AGCTGCTGTAAAACGACGGCCAGT | **B00307** | ACAGAATGTAAAACGACGGCCAGT |
| **B00234** | AGGAAGTGTAAAACGACGGCCAGT | **B00308** | ACAGACTGTAAAACGACGGCCAGT |
| **B00236** | AGGACCTGTAAAACGACGGCCAGT | **B00309** | ACAGAGTGTAAAACGACGGCCAGT |
| **B00237** | AGGACGTGTAAAACGACGGCCAGT | **B00310** | ACAGCATGTAAAACGACGGCCAGT |
| **B00238** | AGGAGATGTAAAACGACGGCCAGT | **B00311** | ACAGCCTGTAAAACGACGGCCAGT |
| **B00239** | AGGAGCTGTAAAACGACGGCCAGT | **B00312** | ACAGCGTGTAAAACGACGGCCAGT |
| **B00240** | AGGAGGTGTAAAACGACGGCCAGT | **B00325** | AGGGAATGTAAAACGACGGCCAGT |
|  |  |  |  |

Additional File 1. The 192 barcoded M13 primers for BAT of amplicons identified to produce clean, single band PCR products.
