# Supplementary material for: Sequencing-based variant detection in the polyploid crop oilseed rape
Source: BMC Plant Biol. 2013 Aug 6;13:111. doi: 10.1186/1471-2229-13-111 (PMC3750413; doi:10.1186/1471-2229-13-111)
Supplement: Additional file 2 — Transcriptome SNP markers assayed using BAT. Word table of primer sequences, SNP details and unigene positions for assayed markers. [file 1471-2229-13-111-S2.docx]

| **Marker** | **SNP** | **AGI** | **F primer** | **Sequence read after F primer - next 35 bases (resolved base marked)** | **Tapidor** | **NingYou7** | **R primer** | **Amplicon size bp (inc barcode)** |
| --- | --- | --- | --- | --- | --- | --- | --- | --- |
| **S00001** | JCVI_21807:127 | AT3G02350.1 | TGTAAAACGACGGCCAGTGTACCGGATCTTCATCTCCG | CCTTGTTCTCTTCCTCTTCCTCGCCACCTTCTCC | T | Y | TGATCCAGGTGAGGTTGGTG | 120 |
| **S00002** | JCVI_19281:407 | AT3G02420.1 | TGTAAAACGACGGCCAGTCCAGAGCACGTGGAGCAG | AATCGGGAGGACGGTGAATCCCATTATCCAACGGT | K | G | TGCATTTTTCTGTTGGTTTTTCACC | 154 |
|  | JCVI_19281:420 |  |  | AATCGGGAGGACGGTGAATCCCATTATCCAACGGT | S | C |  |  |
| **S00003** | EV091127:590 | AT3G02450.1 | TGTAAAACGACGGCCAGTAGCTGTTCCCTCTCCATCC | ATCGTTCCTTACTCAGACTTCGTAACGAATCTTCG | Y | C | AGCAGTGTTGTAATAGATTCG | 142 |
|  | EV091127:593 |  |  | ATCGTTCCTTACTCAGACTTCGTAACGAATCTTCG | Y | T |  |  |
|  | EV091127:602 |  |  | ATCGTTCCTTACTCAGACTTCGTAACGAATCTTCG | M | A |  |  |
| **S00006** | JCVI_27576:692 | AT3G02450.1 | TGTAAAACGACGGCCAGTCGTACATTTGAGAGACGTTCC | TCTAGAAGAAGATGCGTTTCTCATTTGTGATCTGG | Y | T | GAGCAGCGAGCAATGCAGC | 155 |
| **S00007** | EV190755:360 | AT3G02470.1 | TGTAAAACGACGGCCAGTTGTCTTTCTTTGAGCCAAAGC | AATTCTCTGTGGCTGTGCACTCGAGCCTTGGAGCG | C | T | CAAACGTCTGATACATCACCG | 189 |
| **S00008** | JCVI_1429:362 | AT3G02470.1 | TGTAAAACGACGGCCAGTTGTTTTGTTTTGAGCCAAAGC | AATTCTCTGTGGCTGTGCACTCGAGCGTTGGATCG | Y | * | ACTTGCCAAGCTTCTCAAACG | 204 |
| **S00009** | JCVI_10716:253 | AT3G02540.1 | TGTAAAACGACGGCCAGTCAGGTTGCTGAGCCTGTGC | TGGGGGATTTGCTGGCTGCCCACCACTTGCTGGAG | S | G | GGGATCCCCGAGCAAGC | 118 |
|  | JCVI_10716:252 |  |  | TGGGGGATTTGCTGGCTGCCCACCACTTGCTGGAG | G | R |  |  |
| **S00010** | JCVI_9166:343 | AT3G02540.1 | TGTAAAACGACGGCCAGTCTTGGCCATCATAATGACAA | TGAAACTGTTCTCAGCGACCTTGTTCTCCTCAATG | R | G | TAGAGACTGTGCAGGGAGC | 164 |
|  | JCVI_9166:358 |  |  | TGAAACTGTTCTCAGCGACCTTGTTCTCCTCAATG | R | G |  |  |
| **S00012** | JCVI_40139:58 | AT3G02650.1 | TGTAAAACGACGGCCAGTGGGGATCAAACCGTTTTCTG | ATGTGATCCAGAGTTTATGCAGGATGAAGAACGTT | A | R | GTTTCAAACCCCTACTCTCC | 233 |
|  | JCVI_40139:67 |  |  | ATGTGATCCAGAGTTTATGCAGGATGAAGAACGTT | Y | T |  |  |
| **S00014** | JCVI_40139:413 | AT3G02650.1 | TGTAAAACGACGGCCAGTGATTGAAGAGTATGACGAAGC | TCTTAAGCTTTTGAATGAAATGGAGAGTTTCGGTG | R | A | CCCAATCCAATGCTTTGAGG | 146 |
| **S00015** | ES955420:203 | AT3G02660.1 | TGTAAAACGACGGCCAGTTTTTTAATAACTATGACTGGTGG | AAAGATATGACCATGCTGGACTTTCTGAAAAAGGT | Y | C | TGAACAAAGTCATAGCCTTGC | 208 |
| **S00016** | JCVI_27262:1098 | AT3G02690.1 | TGTAAAACGACGGCCAGTCAATGACCCGGTTTTTAACGG | CAGTCTTCAAGAGCTATCCACAAATGATATCATAG | R | * | CCTTTAGTAGCGCTGTAGAAG | 150 |
| **S00018** | JCVI_38601:165 | AT3G02710.1 | TGTAAAACGACGGCCAGTTCGAAGAGCTCGAGAAATCC | TCCCACCACCAAGTCGAAGCCGTCGATCTCAAAAC | R | G | CCGGCGAGGATCTTGAGC | 223 |
| **S00019** | JCVI_124:453 | AT3G02720.1 | TGTAAAACGACGGCCAGTGCCCATAGCCTCTATTTGCC | ACGGGCAGTTGATACTAGCAGCTGCTGACACTGTT | S | G | TGGCCTTGACAAAAAGCTGG | 248 |
| **S00020** | JCVI_2230:170 | AT3G02730.1 | TGTAAAACGACGGCCAGTACTCCGGCGTGGCGACG | ACAACGACGAGGATTGGTTTTTGCTCATTGGATTA | R | A | TTTCACCGGCGGCTTTAACG | 198 |
|  | JCVI_2230:175 |  |  | ACAACGACGAGGATTGGTTTTTGCTCATTGGATTA | S | C |  |  |
|  | JCVI_2230:188 |  |  | ACAACGACGAGGATTGGTTTTTGCTCATTGGATTA | K | T |  |  |
| **S00021** | JCVI_2230:476 | AT3G02730.1 | TGTAAAACGACGGCCAGTGCCATTAGCAAAGGAGCTAG | GAATAAGAGTGGTTCCAACTTTTAAAATATTGAAG | R | A | TCCCGAAGAACTAGCAGACC | 169 |
| **S00022** | JCVI_15987:598 | AT3G02750.1 | TGTAAAACGACGGCCAGTAACCGCAGCACATACATCG | ACTTTTGAGGTTGGGTACTTGTGTCTCCAAGCTCT | * | * | GATGTACTCTCAAATGAAGAGG | 168 |
| **S00023** | JCVI_13535:212 | AT3G02760.1 | TGTAAAACGACGGCCAGTACAAACGGCTTCAAAGATGAC | ACCCGTGTAGTAATCAAGACCTCTAGCTAGACTCA | * | * | AAAGGAGCTCCCATGGAGC | 228 |
| **S00026** | JCVI_4261:682 | AT3G02830.1 | TGTAAAACGACGGCCAGTTTTTCTGGAATGTCCCCCTG | ATGTTTCCACAACCTCATCTGTCTCTGAAGCCGTT | * | T | CTTTTGTGTGTTCTATAGTCGG | 169 |
| **S00029** | JCVI_14866:1353 | AT3G11700.1 | TGTAAAACGACGGCCAGTCCTGAACCGAAATCCGACC | GTCCGTATAAATATCCGGATCAAACAAGTAAGCAG | G | A | CGGAGCAGATAATGTATTACC | 246 |
| **S00030** | JCVI_29858:147 | AT3G11700.1 | TGTAAAACGACGGCCAGTCACAACAAAACCGGGTCTGG | TCAGATAAACTCAAACTCAGTCCTCGTCGCTCTCC | Y | C | GCGTCTGGAGTGATTTGAGG | 268 |
| **S00031** | JCVI_8057:396 | AT3G11710.1 | TGTAAAACGACGGCCAGTTATTCAAACCACCATACTTCTC | AATGTACTCAGGAATAGACATTGACACAGCAAACT | R | G | CTTTGAGAACAGATTGAAATACC | 146 |
| **S00032** | JCVI_7365:589 | AT3G11800.1 | TGTAAAACGACGGCCAGTGCCTCAACAAAGAAGAATGCG | CGATTAAAAGACAAAGCTGCAAGAACCAAGGAGGG | K | G | CAGATTCGCAACCTCGTACC | 173 |
| **S00033** | JCVI_35759:501 | AT3G11820.1 | TGTAAAACGACGGCCAGTTGACGGTGAAGTACCTCCTC | TGTACAGTTTCTCTATACTCAGAGGAGTAAGCTCA | G | R | TGCACGATCCAATATTACACC | 295 |
| **S00034** | JCVI_12505:1768 | AT3G11910.1 | TGTAAAACGACGGCCAGTTTTAGATTTTCATGATAACC | AGAAATGAAATATATAATATCTTTCAGGAAAAAAA | * | * | TGCACGATCCAATATTACACC | 181 |
| **S00035** | EV185171:440 | AT3G11940.1 | TGTAAAACGACGGCCAGTGTGCCCTGAGTTTTACAGTC | TTATCAAAGAAGATCATGTTTTGTTTGGCACTAGA | * | A | ATCTATTCAAAGTAGTTCAGAGG | 158 |
| **S00036** | JCVI_401:577 | AT3G11940.1 | TGTAAAACGACGGCCAGTGAAGAGCCCTTTGCTGCG | TTGATCAATTCATCAGCAAGGCACTCAGCAATAGT | T | Y | GTGGTCCTCGTGAAGATGC | 223 |
|  | JCVI_401:585 |  |  | TTGATCAATTCATCAGCAAGGCACTCAGCAATAGT | R | A |  |  |
|  | JCVI_401:588 |  |  | TTGATCAATTCATCAGCAAGGCACTCAGCAATAGT | G | R |  |  |
| **S00037** | JCVI_499:377 | AT3G11940.1 | TGTAAAACGACGGCCAGTGGATTGGGTTAGCGTCAGTC | AAGAGGTGGATGATTTCCATAGCGTGCTTGATGAT | R | G | CAGTCTTGTTGACTACATTGG | 257 |
| **S00038** | JCVI_12422:239 | AT3G11964.1 | TGTAAAACGACGGCCAGTAAATGCTGGTGGGCCACC | AAAATCTGAATCTTATGACCTGAAGCAATTCCATG | Y | * | GGTCTATTTCAATGAACAAGCC | 143 |
| **S00039** | ES998754:545 | AT3G12060.1 | TGTAAAACGACGGCCAGTAGAAGGCTTGCGTTTGTTGG | AGATTTGCTGAACAGGAACATGTGGGAATCTTTGG | * | * | GTACTCAGCCTCCCAACGG | 165 |
| **S00040** | JCVI_8847:342 | AT3G12260.1 | TGTAAAACGACGGCCAGTCATCATCGGTCAGTACGTGG | TGGGTGAAGGGCTGGTGCAGAATACCGGAAGCAAG | K | G | ACAAGTAGAGAAAACAATCACC | 159 |
|  | JCVI_8847:345 |  |  | TGGGTGAAGGGCTGGTGCAGAATACCGGAAGCAAG | T | Y |  |  |
|  | JCVI_8847:354 |  |  | TGGGTGAAGGGCTGGTGCAGAATACCGGAAGCAAG | S | K |  |  |
| **S00041** | JCVI_1358:224 | AT3G12390.1 | TGTAAAACGACGGCCAGTGGAGAGGCAGGAGGCAAG | TCGAAACAAAGCAGAAGTGAGAAGAAGAGTCGCAA | S | G | CTCTTTTTGACGGTGACACG | 98 |
|  | JCVI_1358:254 |  |  | TCGAAACAAAGCAGAAGTGAGAAGAAGAGTCGCAA | Y | C |  |  |
| **S00042** | JCVI_1358:380 | AT3G12390.1 | TGTAAAACGACGGCCAGTGATGTCTTCAAGAGCCCAGC | ATCAGACACGTACGTCATCTTTGGAGAGGCCAAGA | S | G | GATAGCAGTGACAATATCTCC | 330 |
| **S00044** | JCVI_1358:563 | AT3G12390.1 | TGTAAAACGACGGCCAGTGGATGGTGTTGAGCCAAAGG | ACATTGAGTTGGTGATGACGCAAGCAGGAGTGACT | Y | C | GATAGCAGTGACAATATCTCC | 136 |
| **S00045** | JCVI_636:480 | AT3G12390.1 | TGTAAAACGACGGCCAGTAAGGCTCCGGATCTCAGC | AGCATGATCTCAAAGGGTGAGTCGTCGTCCAGCGC | R | A | GTGGTAAGCTCCATGATGGC | 239 |
|  |  |  |  |  |  |  |  |  |
|  |  |  |  |  | *=undetermined |  |  |  |

Additional File 2. Transcriptome SNP markers assayed using BAT.
