# Supplementary material for: Sequencing-based variant detection in the polyploid crop oilseed rape
Source: BMC Plant Biol. 2013 Aug 6;13:111. doi: 10.1186/1471-2229-13-111 (PMC3750413; doi:10.1186/1471-2229-13-111)
Supplement: Additional file 3 — Linkage mapping of SNP markers scored by BAT method. Word table of mapping positions of SNP markers from genotyping determined by GoldenGate and BAT scoring and read depth details for BAT SNP scoring. [file 1471-2229-13-111-S3.docx]

| **Marker** | **SNP** | **AGI** | **Linkage group** | **Reads** | | **Average read depth per barcode** | |  |
| --- | --- | --- | --- | --- | --- | --- | --- | --- |
|  |  |  |  |  | |  | |  |
| **S00003** | EV091127:590 | AT3G02450.1 | C5 | 11,731 | | 244 | |  |
| **S00003** | EV091127:593 | AT3G02450.1 | C5 | 11,731 | | 244 | |  |
| **S00006** | JCVI_27576:692 | AT3G02450.1 | C5 | 17,838 | | 372 | |  |
| **S00007** | EV190755:360 | AT3G02470.1 | A3 | 504 | | 11.2 | |  |
| **S00008** | JCVI_1429:362 | AT3G02470.1 | A3 | 184 | | 5.3 | |  |
| **S00009** | JCVI_10716:252 | AT3G02540.1 | A3 | 10,504 | | 219 | |  |
| **S00009** | JCVI_10716:253 | AT3G02540.1 | A3 | 10,504 | | 219 | |  |
| **S00010** | JCVI_9166:343 | AT3G02540.1 | A3 | 21,518 | | 448 | |  |
| **S00010** | JCVI_9166:358 | AT3G02540.1 | A3 | 21,518 | | 448 | |  |
| **S00012** | JCVI_40139:58 | AT3G02650.1 | C1 | 6,589 | | 146 | |  |
| **S00012** | JCVI_40139:67 | AT3G02650.1 | C1 | 6,589 | | 146 | |  |
| **S00014** | JCVI_40139:100 | AT3G02650.1 | C1 | 7,776 | | 162 | |  |
| **S00015** | ES955420:203 | AT3G02660.1 | C1 | 7,747 | | 161 | |  |
| **S00019** | JCVI_124:453 | AT3G02720.1 | A5 | 35,689 | | 743 | |  |
| **S00030** | JCVI_29858:147 | AT3G11700.1 | A5 | 16,422 | | 342 | |  |
| **S00031** | JCVI_8057:396 | AT3G11710.1 | A1 | 6,534 | | 139 | |  |
| **S00032** | JCVI_7365:589 | AT3G11800.1 | A3 | 4,291 | | 89 | |  |
| **S00033** | JCVI_35759:501 | AT3G11820.1 | A5 | 4,098 | | 85 | |  |
| **S00033** | JCVI_35759:504 | AT3G11820.1 | A5 | 4,098 | | 85 | |  |
| **S00037** | JCVI_499:377 | AT3G11940.1 | A5 | 29,153 | | 607 | |  |
| **S00040** | JCVI_8847:342 | AT3G12260.1 | A1 | 11,655 | | 243 | |  |
| **S00040** | JCVI_8847:345 | AT3G12260.1 | A1 | 11,655 | | 243 | |  |
| **S00040** | JCVI_8847:354 | AT3G12260.1 | A1 | 11,655 | | 243 | |  |
| **S00044** | JCVI_1358:563 | AT3G12390.1 | A1 | 7,207 | | 150 | |  |
|  |  |  |  |  | |  | |  |
| AGI: Arabidopsis Gene Initiative gene model code | | | | |  | |  | |

Additional File 3. Linkage mapping of SNP markers scored by BAT method.
