# Supplementary material for: Sequencing-based variant detection in the polyploid crop oilseed rape
Source: BMC Plant Biol. 2013 Aug 6;13:111. doi: 10.1186/1471-2229-13-111 (PMC3750413; doi:10.1186/1471-2229-13-111)
Supplement: Additional file 5 Table S5 — Barcode assignment for SNP transcriptome marker genotyping using BAT. Word table containing individual population line barcode assignments. [file 1471-2229-13-111-S5.docx]

| **TNDH Line :** | **Barcode :** | **Sequence** |
| --- | --- | --- |
| 004 | B00002 | AACAAGTGTAAAACGACGGCCAGT |
| 005 | B00003 | AACACATGTAAAACGACGGCCAGT |
| 008 | B00004 | AACACCTGTAAAACGACGGCCAGT |
| 010 | B00006 | AACAGATGTAAAACGACGGCCAGT |
| 012 | B00007 | AACAGCTGTAAAACGACGGCCAGT |
| 016 | B00009 | AACATATGTAAAACGACGGCCAGT |
| 017 | B00010 | AACATCTGTAAAACGACGGCCAGT |
| 018 | B00011 | AACATGTGTAAAACGACGGCCAGT |
| 024 | B00012 | AACCAATGTAAAACGACGGCCAGT |
| 028 | B00013 | AACCACTGTAAAACGACGGCCAGT |
| 031 | B00014 | AACCAGTGTAAAACGACGGCCAGT |
| 033 | B00015 | AACCGATGTAAAACGACGGCCAGT |
| 038 | B00016 | AACCGCTGTAAAACGACGGCCAGT |
| 044 | B00018 | AACCTATGTAAAACGACGGCCAGT |
| 047 | B00019 | AACCTCTGTAAAACGACGGCCAGT |
| 048 | B00020 | AACCTGTGTAAAACGACGGCCAGT |
| 051 | B00021 | AACGAATGTAAAACGACGGCCAGT |
| 057 | B00022 | AACGACTGTAAAACGACGGCCAGT |
| 066 | B00023 | AACGAGTGTAAAACGACGGCCAGT |
| 072 | B00024 | AACGCATGTAAAACGACGGCCAGT |
| 076 | B00025 | AACGCCTGTAAAACGACGGCCAGT |
| 077 | B00027 | AACGGATGTAAAACGACGGCCAGT |
| 085 | B00030 | AACGTCTGTAAAACGACGGCCAGT |
| 088 | B00031 | AACGTGTGTAAAACGACGGCCAGT |
| 090 | B00032 | AACTAATGTAAAACGACGGCCAGT |
| 103 | B00033 | AACTACTGTAAAACGACGGCCAGT |
| 108 | B00034 | AACTAGTGTAAAACGACGGCCAGT |
| 109 | B00035 | AACTCATGTAAAACGACGGCCAGT |
| 121 | B00036 | AACTCCTGTAAAACGACGGCCAGT |
| 124 | B00037 | AACTCGTGTAAAACGACGGCCAGT |
| 126 | B00038 | AACTGATGTAAAACGACGGCCAGT |
| 128 | B00039 | AACTGCTGTAAAACGACGGCCAGT |
| 129 | B00040 | AACTGGTGTAAAACGACGGCCAGT |
| 135 | B00042 | AAGAAGTGTAAAACGACGGCCAGT |
| 138 | B00043 | AAGACATGTAAAACGACGGCCAGT |
| 145 | B00045 | AAGACGTGTAAAACGACGGCCAGT |
| 149 | B00046 | AAGAGATGTAAAACGACGGCCAGT |
| 158 | B00047 | AAGAGCTGTAAAACGACGGCCAGT |
| 160 | B00048 | AAGAGGTGTAAAACGACGGCCAGT |
| 161 | B00051 | AAGCAGTGTAAAACGACGGCCAGT |
| 163 | B00052 | AAGCCATGTAAAACGACGGCCAGT |
| 170 | B00054 | AAGCGATGTAAAACGACGGCCAGT |
| 171 | B00057 | AAGGAATGTAAAACGACGGCCAGT |
| 172 | B00060 | AAGGCATGTAAAACGACGGCCAGT |
| 176 | B00063 | AAGTAATGTAAAACGACGGCCAGT |
| 177 | B00064 | AAGTACTGTAAAACGACGGCCAGT |
|  |  |  |

Additional File 4. Barcode assignment for SNP transcriptome marker genotyping using BAT.
