# Supplementary material for: Sequencing-based variant detection in the polyploid crop oilseed rape
Source: BMC Plant Biol. 2013 Aug 6;13:111. doi: 10.1186/1471-2229-13-111 (PMC3750413; doi:10.1186/1471-2229-13-111)
Supplement: Additional file 9 — BAT mutation detection amplicons. Amplified sequences are shown for Bna.FAD2 family (panel a) and BnaC.GL2.b (panel b). Regions of the amplicons are colour-coded: barcode (blue), M13 (red) and specific primer (green) positions. Regions of the amplicons that can be re-sequenced with 76 base reads, as used in the experiments, are not coloured. Regions not covered by the 76 bp read are coloured grey. Description: Illustration of sequence regions covered within the BAT mutation screen. [file 1471-2229-13-111-S9.docx]

a

b

Additional File 8. BAT mutation detection amplicons. Amplified sequences are shown for *BnaFAD2* family (panel a) and *BnaC.GL2.b* (panel b). Regions of the amplicons are colour-coded: barcode (blue), M13 (red) and specific primer (green) positions. Regions of the amplicons that can be re-sequenced with 76 base reads, as used in the experiments, are not coloured. Regions not covered by the 76bp read are coloured grey.
