# Supplementary material for: Sequencing-based variant detection in the polyploid crop oilseed rape
Source: BMC Plant Biol. 2013 Aug 6;13:111. doi: 10.1186/1471-2229-13-111 (PMC3750413; doi:10.1186/1471-2229-13-111)
Supplement: Additional file 11 — Bna.FAD2 coamplification and BnaC.GL2.b-specific primers used to produce the amplicons for BAT mutation screening. Word table containing primer details of screened amplicons. [file 1471-2229-13-111-S11.docx]

| **Primer** | **Sequence (5'-3')** |
| --- | --- |
|  |  |
| **BnaFAD2BATf** | TGTAAAACGACGGCCAGTCCCATCTACAACGACCAY |
| **BnaFAD2BATr** | TCMACRGTRGCCAAAGCTCC |
| **BnaGL2bBATf** | ATGTGTGTATCTGTGCAGGAG |
| **BnaGL2bBATr** | TGTAAAACGACGGCCAGTGTTATGACTAAAGGTTATTAGTACCC |
|  |  |

Additional File 10. *Bna.FAD2* coamplification and BnaC.GL2.b specific primers used to produce the amplicons for BAT mutation screening.
