# Supplementary material for: Sequencing-based variant detection in the polyploid crop oilseed rape
Source: BMC Plant Biol. 2013 Aug 6;13:111. doi: 10.1186/1471-2229-13-111 (PMC3750413; doi:10.1186/1471-2229-13-111)
Supplement: Additional file 12 — Primers used for the amplification of Bna.FAD2 loci. Word table containing primers used for the amplification of Bna.FAD2. [file 1471-2229-13-111-S12.docx]

| **Primer No.** | **Sequence (5'-3')** | **Length (bp)** | **GC %** | **Tm** |
| --- | --- | --- | --- | --- |
|  |  |  |  |  |
| 1 | GTCTCCTCCCTCCAAAAAGT | 20 | 50.0 | 54.9 |
| 2 | GTGTCTCCTCCCTCCAAA | 18 | 55.6 | 51.9 |
| 3 | CTACAGAAACAAACATGGGC | 20 | 45.0 | 53.1 |
| 4 | CTCTCCTCCCTCCAGCTCCC | 20 | 70.0 | 62.4 |
| 5 | CTCTTCGACATCCTCCTCTC | 20 | 55.0 | 53.3 |
| 6 | CCTCGTCCCTTACTTCTCCTG | 21 | 57.1 | 58.2 |
| 7 | CCTCATAACTTATTGTTGTACCAG | 24 | 37.5 | 53.9 |
| 8 | CAAGACGACCAGAGACAGC | 20 | 55.3 | 55.0 |
| 9 | GAACTCGACAAATTTGCCTG | 20 | 55.7 | 45.0 |
|  |  |  |  |  |

Additional File 11. Primers used for the amplification of *Bna.FAD2.*
