# Supplementary material for: Sequencing-based variant detection in the polyploid crop oilseed rape
Source: BMC Plant Biol. 2013 Aug 6;13:111. doi: 10.1186/1471-2229-13-111 (PMC3750413; doi:10.1186/1471-2229-13-111)
Supplement: Additional file 13 — Primer combinations required for the locus-specific amplification of FAD2 orthologues. Word table containing primer combinations. [file 1471-2229-13-111-S13.docx]

| **Homologue** | **F primer** | | **R primer** | | **Amplicon size (bp)** | | | **Notes** | |
| --- | --- | --- | --- | --- | --- | --- | --- | --- | --- |
|  | |  | |  | |  |  | |  |
| *Bna*C.*FAD2*.a | | 1 | | 8 | | 1212 |  | |  |
| *BnaA*.*FAD2*.a | | 2 | | 7 | | 1133 |  | |  |
| *Bna*C.*FAD2*.b | | 5 | | 7 | | 991 | Will not amplify in Cabriolet | |  |
| *BnaA*.*FAD2*.b | | 6 | | 9 | | 966 |  | |  |
| *BnaA*.*FAD2*.b | | 3 | | 7 | | 1173 | *Bna*A.*FAD2*.a-specific in Cabriolet. Will also amplify *Bna*C.*FAD2*.a in other genotypes. | |  |
| *BnaA*.*FAD2*.b | | 4 | | 7 | | 1133 | *Bna*A.*FAD2*.a-specific in Cabriolet. Will also amplify *Bna*C.*FAD2*.a in other genotypes. | |  |
|  | |  | |  | |  |  | |  |

Additional File 12. Primer combinations required for the locus-specific amplification of *FAD2* orthologues.
